# A Review on the Use of Antimicrobial Peptides to Combat Porcine Viruses

**DOI:** 10.3390/antibiotics9110801

**Published:** 2020-11-12

**Authors:** Guihong Pen, Na Yang, Da Teng, Ruoyu Mao, Ya Hao, Jianhua Wang

**Affiliations:** 1 Gene Engineering Laboratory, Feed Research Institute, Chinese Academy of Agricultural Sciences, Beijing 100081, China; pgh1316191@163.com (G.P.); tengda@caas.cn (D.T.); maoruoyu@caas.cn (R.M.); haoya@caas.cn (Y.H.); 2 Key Laboratory of Feed Biotechnology, Ministry of Agriculture and Rural Affairs, Beijing 100081, China

**Keywords:** antimicrobial peptides, antiviral, swine, mechanism of action

## Abstract

Viral infectious diseases pose a serious threat to animal husbandry, especially in the pig industry. With the rapid, continuous variation of viruses, a series of therapeutic measures, including vaccines, have quickly lost their efficacy, leading to great losses for animal husbandry. Therefore, it is urgent to find new drugs with more stable and effective antiviral activity. Recently, it has been reported that antimicrobial peptides (AMPs) have great potential for development and application in animal husbandry because of their significant antibacterial and antiviral activity, and the antiviral ability of AMPs has become a research hotspot. This article aims to review the research situation of AMPs used to combat viruses in swine production of animal husbandry, clarify the mechanism of action of AMPs on viruses and raise some questions, and explore the future potential of AMPs in animal husbandry.

## 1. Introduction

Viral infection of pigs is one of the bottlenecks restricting the development of the pig industry globally. The widely spread common porcine pathogenic viruses, including herpesvirus (such as pseudorabies virus (PRV)), coronavirus (such as porcine epidemic diarrhea virus (PEDV)), and arterivirus (such as porcine reproductive and respiratory syndrome virus (PRRSV)), have caused many serious infectious diseases and huge economic losses in the pig industry [1,2,3]. At present, only a few effective treatments are available for most viral diseases. In the past few decades, research on fighting viral infections in pigs has been mainly focused on vaccines, wherein the adaptive immunity of pigs is improved by vaccination [4,5]. However, some viruses can escape from host immunity through different strategies. It is reported that PRRSV evades the host immune response by glycosylation modification of its envelope proteins [6], and PEDV evades the host innate immune response by encoding interferon (IFN) antagonists to disrupt the innate immune pathway and hide its viral RNA to avoid exposure of viral RNA to immune sensors [7]. The emergence of new variants of viruses is one of the important causes of disease outbreaks in pigs, such as the reemergence of PRV in China since late 2011 [8], the outbreak of porcine epidemic diarrhea (PED) in China at the end of 2010 [9], and the spread of African swine fever worldwide since 2007 [10]. Besides, the development of new vaccines is usually complex, technically challenging, and time-consuming [11]. Therefore, there is an urgent need to develop novel effective agents to kill viruses and prevent their infection in swine [4].

Antimicrobial peptides (AMPs) are small proteins with potential activity against, for example, bacteria, viruses, fungi, tumors, and parasites, which are widely found in animals, plants, and microorganisms [12]. The discovery of AMPs can be traced back to 1939; Dubos isolated an antibacterial agent from a strain of soil *Bacillus* and found that it could protect mice from pneumococcal infection for the first time [13,14]. According to their key structural characteristics, AMPs can be usually divided into four categories: α-helix AMPs, β-folded AMPs, extended structural AMPs, and cyclic structural AMPs [15]. Since their discovery, AMPs have become important alternative drugs in the field of disease prevention and immune regulation, attracting worldwide attention [16,17]. In the field of human viral diseases, at present, AMPs have become an important direction and field in antiviral research. Previous studies have shown that the amphibian-derived AMPs, caerin 1.1 and maculatin 1.1, completely inhibited human immunodeficiency virus (HIV) [18]; arthropod-derived AMPs, cecropin A and melittin, could effectively inhibit Junin virus (JV) multiplication and impede the multiplication of herpes simplex virus (HSV) and JV, respectively [19]; plant protein kalata B1 analogs could inhibit dengue virus (DENV) [20]. The above results suggested that AMPs with antiviral activity have the potential to be antiviral drugs and could be highly expected to become clinical drugs for both human viral diseases and animal viral infections. However, there have only been a few studies on AMPs in animals compared with humans so far. It is important to pay attention to the role of AMPs in animal health because most upstream studies specialized for human usage are run with model animals.

**Figure 1:**
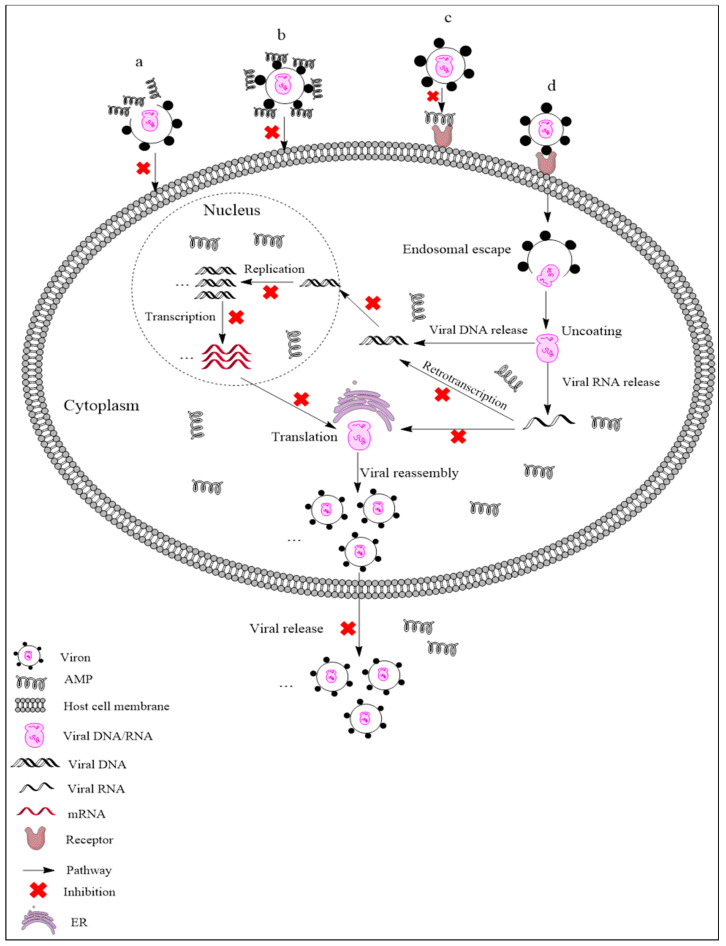
Antiviral mechanisms of AMPs. (**a**) AMPs inactivate a virus by destroying the viral envelope. (**b**) AMPs bind to glycoprotein on the viral envelope. (**c**) AMPs occupy a specific receptor on the host cell membrane to prevent viral attachment. (**d**) AMPs inhibit viral replication, transcription, reverse transcription, translation, and release.

AMPs exhibit different mechanisms on viruses, and their main antiviral mechanisms and types are summarily shown in Figure 1. (i) AMPs neutralize viruses by integrating into the viral envelope or host cell membranes, and both enveloped RNA and DNA viruses can be targeted [21,22]. Indolicidin could inactivate human immunodeficiency virus (HIV) by binding to the envelope and cracking the membrane through a membrane splitting mechanism, thereby preventing the virus from infecting the host cell [23]. (ii) AMPs bind to glycoproteins on the virus surface to inhibit viral infection. Defensin retrocyclin 2 bound to immobilized herpes simplex virus type 2 (HSV-2) glycoprotein B with high affinity so that HSV-2 could not bind to the surface of host cells [24]. (iii) AMPs can interact with specific receptors of host cell membranes, preventing virus particles from binding to host cells. HSV particles infect host cells by binding to heparan sulfate on the host cells, while the α-helix cationic polypeptide lactoferrin can prevent HSV infection by occupying heparan sulfate [25]. (iv) In addition to the above points, AMPs may also act on other stages of the viral life cycle. For example, human beta-defensin-3 (hBD-3) inhibits HIV replication by acting on entry, reverse transcription, and nuclear import of retroviral DNA [26]. Besides, some AMPs, such as cecropin D (CD), could block apoptosis induced by PRRSV at the late stage of infection, thus inhibiting the assembly, release, and transmission of the virus [27].

As the pig industry has developed to account for the largest proportion of food animals in China, AMPs have been widely studied as antimicrobial agents and/or feed additives [28,29,30,31]. Studies have shown that the plectasin-derived peptides NZ2114 and MP1102 could effectively kill *Streptococcus suis* type 2 [28,29], and NZX exhibited antibacterial activity against *Staphylococcus hyicus* [30]. Diets supplemented with AMPs could improve the growth performance, nutrition maintenance, intestinal morphology, and immunity of weaned piglets, while also reducing the presence of harmful microorganisms in these animals [31]. In addition to antibacterial activity and immune regulation, AMPs should also be actively encouraged and utilized to combat common porcine pathogenic viruses in pig molecular breeding via genetic engineering. The purpose of this article is mainly to review the research status of AMPs combating viruses in swine production, clarify the mechanism of action of AMPs on viruses, and explore the future potential of AMPs as antiviral drugs in animal husbandry.

## 2. Antimicrobial Peptides Used against Viruses in Swine

### 2.1. AMPs Active against PRV and PEDV

PRV is a large, enveloped, double-stranded DNA virus that is the pathogen of Aujeszky’s disease and belongs to the porcine neurotropic herpesviruses [1,32]. Clinically, it is characterized by severe neurological disorders in newborn piglets and reproductive disorders in sows [32]. PEDV is also an enveloped virus; it is a positive-sense, single-stranded RNA virus, belonging to the family of *Coronaviridae* [2]. PEDV is the pathogen of porcine epidemic diarrhea (PED), which is an acute infectious enteropathy and is characterized by severe watery diarrhea, vomiting, and dehydration of suckling piglets, causing huge economic losses for the pig industry [33].

#### 2.1.1. Piscidin-1

**Figure 2:**
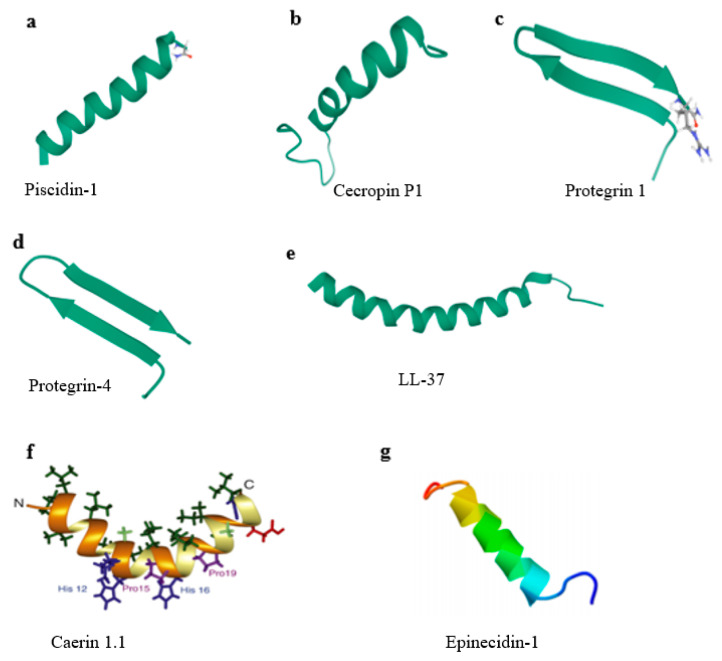
Structure types of typical AMPs. (**a**) Solid-state NMR structure of piscidin-1 in aligned 1:1 phosphatidylethanolamine/phosphoglycerol lipid bilayers (PDB ID 2MCV). (**b**) Solution structure of cecropin P1 with LPS (PDB ID 2N92). (**c**) Protegrin 1 from porcine leukocytes, NMR, 20 structures (PDB ID 1PG1). (**d**) Structure of protegrin-4 by high-resolution NMR spectroscopy (PDB ID 6QKF). (**e**) Structure of human LL-37 (PDB ID 2K6O). (**f**) Solution structures of caerin 1.1 [56]. (**g**) Structure of epinecidin-1 [57].

Piscidin-1, a natural polypeptide, is produced by mast cells of fish, belongs to the piscidin family, and is characterized by a conserved N-terminus rich in histidine and phenylalanine and a variable C-terminus [34,35]. Piscidin-1 consists of 22 amino acids (Table 1) and shows an α-helix conformation (Figure 2a) which is the best among its family members in terms of antimicrobial activity towards multiple pathogenic bacteria [36]. In addition, piscidin-1 has a potent effect on viruses such as catfish virus (CCV), frog virus 3 (FV3), and HIV-1 [37,38]. Lei et al. demonstrated that piscidin-1 displays a significant inhibitory effect on PRV and PEDV [39]. The plaque elimination rates were reached as high as 95% (PRV) and 85% (PEDV) at 1 and 2 µg/mL piscidin-1, respectively, as determined by plaque reduction assay. Moreover, in a pharmacokinetic study administering 2 mg/kg piscidin-1 by three drug delivery routes (intravenous injection (i.v.), intramuscular injection (i.m.), and per os (p.o.)) in rat, the highest bioavailability (73.17%) of piscidin-1 was observed for i.m. administration; therefore, i.m. represents the best pathway in potential drug delivery when piscidin-1 is used to inhibit PEDV in vivo [39]. Piscidin-1 could effectively protect mice from PRV infection when PRV was co-injected along with piscidin-1 at the concentration above 5 μg/mL, and the in vivo protection rate was still as high as 90% at a low piscidin-1 concentration of 2.5 μg/mL [40]. These results indicate that piscidin-1 may directly interact with PRV particles and block cell apoptosis induced by PRV [40]. In addition, it was demonstrated that piscidin-1 also has inhibitory effects on several common porcine pathogenic viruses, including PRRSV, PEDV, transmissible gastroenteritis virus (TGEV), and rotavirus (RV) [40].

#### 2.1.2. Caerin 1.1

Caerin 1.1 is an AMP that consists of 25 amino acid residues (Table 1) and is derived from granular glands in the skin of the Australian tree frog [41]. Nuclear magnetic resonance (NMR) shows that caerin 1.1 has two α-helices and a flexible hinge region; moreover, the hinge region contains two proline residues (Figure 2f) [56], which play an important role in the mechanism of antimicrobial action [58]. Caerin 1.1 shows activity against bacteria and viruses, destroying the integrity of pathogen particles by forming holes in the membrane [18,59]. Guo et al. revealed that caerin 1.1 shows low cytotoxicity and exhibits excellent anti-PEDV activity in a dose-dependent manner even at low concentrations (2.5, 5, 10, and 20 µg/mL). The study indicated that caerin 1.1 suppresses the growth of PEDV in vitro via direct binding to the viruses, destroys the structure of viral particles, and decreases the titers of viruses almost up to 3 logs [60]. In addition, caerin 1.1 also blocks the release of PEDV particles during virus replication to control the infection process, thus reducing the transmission of virus between neighboring cells [60]. Hu et al. showed that caerin 1.1 has a potent anti-PEDV activity that resulted in the residual infectivity being 0.2% in the TCID50 assay [40]. Besides, caerin 1.1 could not only inhibit most of the PRV particles by directly interacting with PRV but also block host cell apoptosis at the concentration of 25 µg/mL, sharing the same antiviral mechanism as piscidin-1. In addition, caerin 1.1 also inhibits the infection of PRRSV, TGEV, and RV [40].

#### 2.1.3. Porcine β-Defensin-2 (pBD-2)

Defensins are a group of cationic antibacterial peptides consisting of 18–45 amino acids forming three intramolecular disulfide bonds between conserved cysteine residues [61]. Based on their spatial structure and disulfide linkage, defensins are divided into α-, β-, and θ-defensins in vertebrates [62]. The β-defensin family is mainly expressed in epithelial cells of animal skin, respiratory tract, and gastrointestinal tract [63]. At present, more than 30 β-defensins have been found in humans, but only one β-defensin has been reported in pigs. Porcine β-defensin-2 (pBD-2) is highly expressed in epithelial cells of pigs; it is a cysteine-rich cationic antibacterial peptide composed of 37 amino acid residues (Table 1), showing the characteristics of antibacterial activity, immunoregulation, and intestinal tract protection [42,43,64,65]. In addition, pBD-2 is also reported to have antiviral activity. Huang et al. confirmed the antiviral ability of pBD-2 both in vitro and in vivo. In their research, pBD-2 inhibited PRV proliferation at a threshold concentration (40 μg/mL) and displayed no significant cytotoxicity towards PK-15 cells even at a maximum concentration (80 μg/mL) [66]. The results suggested that pBD-2 has a direct killing effect against PRV by destroying the viral envelopes and also affects PRV entry into host cells. However, further research is necessary to determine whether pBD-2 can block virus binding and infiltration by interacting with specific receptors in the host membrane [66].

### 2.2. AMPs Active against PRRSV

Porcine reproductive and respiratory syndrome (PRRS), also known as blue-ear pig disease, is one of the most fatal infectious diseases in the pig industry around the world; it was first reported in North America and Canada in the late 1980s [67,68,69]. The pathogen of this disease is called the porcine reproductive and respiratory syndrome virus (PRRSV), which is an enveloped, single-stranded RNA virus belonging to the *Arteriviridae* family of *Nidovirales*. Its genomic RNA length is about 15.4 kb, with a 5′ cap, 3′ polyadenylation, and 10 open reading frames (ORFs) [3,70]. The main manifestations of the disease are poor reproductive performance and high miscarriage rate in pregnant sows and dyspnea in growing–finishing pigs and piglets [71]. PRRSV mainly infects porcine alveolar macrophages (PAMs) and has the characteristics of high mutation rate and high recombination rate. With the antigen variation and genetic drift of the virus, the existing vaccines are easily losing their efficacy [72,73]. Therefore, PRRSV is still the greatest challenge facing the pig industry so far, and it is urgent to develop new antiviral strategies to combat PRRSV infection [74].

#### 2.2.1. Cecropins

Cecropins are small molecules with a size ranging from 3 to 4 kDa and are considered to be the one of most typical AMPs with a long history [75]. Structurally, cecropins have a strongly basic amino (N)-terminal and a hydrophobic carboxyl (C)-terminal [76].

Cecropin D (CD) was first isolated from the pupa of *Hyalophora cecropia* [77]. CD consists of 36 amino acid residues (Table 1) without cysteine and mainly shows a helical conformation [44]. CD exhibits homology with cecropin A and cecropin B. Recombinant CD has been successfully expressed in *Pichia pastoris* and has antibacterial activity against both Gram-positive and Gram-negative bacteria [75]. CD shows no significant cytotoxicity at the concentration of 300 mg/L, and it was found to effectively inhibit HP-PRRSV strain Li11 both in Marc-145 cells and PAMs in in vitro experiments. In brief, the mechanisms of CD against viruses were tracked via the following multiple pathways: (i) blocking the attachment of PRRSV to the membrane of Marc-145 cells to inhibit virus entry and replication, thus reducing the generation of progeny virus; (ii) inhibiting virus RNA transcription and viral protein expression (i.e., the transcription activity of Li11 gene in terms of its mRNA content was highly inhibited with a treatment of 300 mg/L CD); (iii) attenuating apoptosis induced by PRRSV at the late stage of infection and suppressing the release of viral particles [27].

Cecropin P1 (CP1) is produced by parasitic nematode *Ascaris suum* from the intestinal tract of pigs and composed of 31 amino acid residues (Table 1), and it is highly similar to two insect cecropins (Cecropin B and Cecropin IA) [45,78]. CP1 has a continuous amphipathic α-helical structure (Figure 2b) and the helix can easily span a bacterial lipid membrane [79,80]. CP1 was found to inhibit PRRSV infection with a 50% effective concentration (EC_50_) of 112 μg/mL in Marc-145 cells and 65 μg/mL in PAMs, showing no significant cytotoxicity when the concentration reached 480 μg/mL in Marc-145 cells. Moreover, the results proved that CP1 potently prevents the replication of PRRSV at multiple points in the viral life cycle. On one hand, CP1 inhibits the synthesis of RNA and protein and the release of virus particles in Marc-145 cells; on the other hand, it weakens the apoptosis induced by the virus in the later stage of infection [46]. The molecular mechanism of CP1 was further investigated by binding and entry assays in Marc-145 cells, and the results showed that CP1 prevents viral adsorption during the viral life cycle and thus disrupts the initial step of viral entry into the target cells, suggesting that CP1 blocks the interaction between PRRSV and its receptors on the cell membrane [46] and effectively inhibits virus infection.

#### 2.2.2. Host Defense Peptides (HDPs)

There are two main families of host defense peptides (HDPs) in mammals: defensins and cathelicidins. Defensins are mainly distributed in the epithelial cells and phagocytes of mammals, while cathelicidins are mainly derived from neutrophils in blood and expressed on epithelial surfaces.

**Figure 3:**
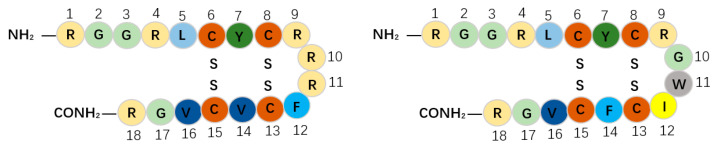
Sequences of PG-1 and PG-4. The same amino acids are represented by the same color. PG-4 substitutes a phenylalanine for a valine at position 14, and their sequences are different in the β-turn.

Protegrins, as members of the porcine cathelicidins family, were first discovered in porcine leukocytes; they combine the features of corticostatic defensins and tachyplesins. Protegrins contain 16–18 amino acid residues and show potential activity towards some pathogenic enveloped viruses, bacteria, and fungi [47]. There are five porcine protegrins, namely PG-1 to PG-5; among them, PG-1 and PG-4 both contain 18 amino acid residues stabilized with two cysteine bridges by four cysteine residues (Table 1) [47,49] and have an anti-parallel β-strand structure (Figure 2c,d) [81]. It was initially reported that PG-1 could inhibit both HP-PRRSV strain Li10 and N-PRRSV strain CH-1a infection at the concentrations of 20–40 mg/L and inhibit Li10 replication at the concentrations of 30–40 mg/L without cytotoxicity in Marc-145 cells [50]. The results suggested that PG-1 (40 mg/L) can block the release of Li10 particles and partially block Li10 internalization. It is reported that PG-1 might disturb the interactions between viral particles and cell-membrane receptors and thus block the attachment stage of the virus at the early period. However, later research noted that PG-1 can neither inhibit Li10 replication nor elevate antiviral cytokine expression in PAMs [50]. In comparison with PG-1, PG-4 substitutes a phenylalanine for a valine at position 14, and their sequences are different in the β-turn (Figure 3). Sang et al. showed that PG-4 could suppress PRRSV in PAMs at the concentrations of 5–40 μg/mL, indicating that the activity of PG-4 is much higher than that of PG-1; this finding suggests that the aromatic side chain of phenylalanine plays an important role in activity against PRRSV [48].

Porcine β-defensin-2 (pBD-2), which was described in a previous section, can inhibit not only PRV but also PRRSV. Veldhuizen et al. indicated that pBD-2 can inhibit the proliferation of PRRSV in MA-104 cells when its concentration reaches 64 μg/mL [42]. In addition, porcine β-defensin-3 (pBD-3) consists of 38 amino acid residues (Table 1). pBD-3, like pBD-2, is mainly expressed in bone marrow, liver, lung, skin, and other lymphoid tissues like pBD-2 [43]. A study showed that pBD-3 consistently suppressed PRRSV titers in PAMs at the concentrations of 5–40 μg/mL. The precise mechanism by which pBD-3 inhibits PRRSV infection remains to be determined [48].

Human cathelicidin LL37, consisting of 37 amino acid residues (Table 1), adopts a long helix covering residues 2–31 with a disordered C-terminal tail (Figure 2e) [51]. LL37 not only possesses antibacterial, antifungal, antiviral, and anticancer functions but also shows an immunoregulatory effect and even exhibits a potential activity of promoting wound repair, apoptosis, and angiogenesis regulation [82]. A previous study showed that cathelicidin LL37 displays direct antiviral activity instead of inducing apoptosis or necrosis [83]. Levast et al. indicated that LL37 shows anti-PRRSV activity by reducing viral replication in vitro, and it might enter into PRRSV and directly interact with nucleic acid to inhibit its replication, but further studies are still needed to fully reveal the exact anti-PRRSV mechanism of LL37 in detail [52].

### 2.3. Epinecidin-1 (Epi-1) Fights against FMDV

Foot and mouth disease (FMD) is a very important disease affecting livestock in the world [84] and is caused by foot and mouth disease virus (FMDV) [85]. FMDV is a nonenveloped virus belonging to the family of *Picornaviridae* and genus *Aphthovirus* [86]; it is highly infectious to pigs and other cloven-hoofed animals and imposes a significant impact on the global economy [87,88]. Common symptoms of foot and mouth disease include fever and blistering lesions in the mouth, tongue, and feet. There are seven antigenic serotypes of FMDV, including O, A, C, SAT1, SAT2, SAT3 (South African 1, 2, 3), and Asia1 (Asian 1); each serotype has multiple subtypes [89], and serotype O is the most common serotype in the world. As there is still no effective vaccine or antiviral drug, new and better drugs or candidates are being sought to combat FMDV infection.

Epinecidin-1 (Epi-1) is derived from the orange-spotted grouper, *Epinephelus coioides* [90], and belongs to the piscidin peptide family. The piscidin family is an evolutionarily conserved, linear, amphiphilic, antibacterial peptide family that is unique to fish and homologous to cecropins [91]. The length of complete Epi-1 cDNA is 518 base pairs, and the longest open reading frame consists of 204 base pairs and encodes a sequence of 67 amino acids [90]. Studies on the potential pharmacological activity have mainly focused on amino acid residues 22–42 of Epi-1 (Table 1) [53], which shows an α-helical structure without disulfide bonds (Figure 2g) [57]. Epi-1 has been reported to have wide activity against bacteria, fungi, and viruses and shows immune regulation [92,93,94]. Besides, Huang et al. found that the synthetic Epi-1 effectively suppresses FMDV (type O/Taw/97) by inactivating virus particles and inhibiting virus proliferation. Epi-1 not only shows a direct antiviral effect on FMDV at high concentration (10 × EC_90_ concentration of 125 μg/mL) but also prevents the adsorption of FMDV on BHK-21 cells at low concentration (6.2 μg/mL) [54]. Since a structured membrane is absent in FMDV, research data indicated that the application of Epi-1 in virus adsorption can effectively inhibit virus replication, and thus it is suggested that Epi-1 could interfere with the early stage of viral infection through an undisclosed mechanism [54].

### 2.4. Synthesized Peptides Fight against ASFV

In addition to the above several viral diseases, African swine fever (ASF) is also a viral disease that lacks effective vaccines for prevention and control in the pig breeding industry; it has spread quickly as an epidemic viral disease in China since mid-2018. As a highly infectious viral disease of pigs, ASF causes fatal hemorrhagic fever after infection, resulting in a high mortality rate of nearly 100% [95]. ASF is caused by African swine fever virus (ASFV), which is a large, enveloped, double-stranded DNA virus with icosahedral morphology and the only member of *Asfarviridae* family. ASFV is transmitted by arthropod soft ticks (*Ornithodoros moubata*), making it the only DNA virus to be transmitted via insect [96,97].

**Figure 4:**
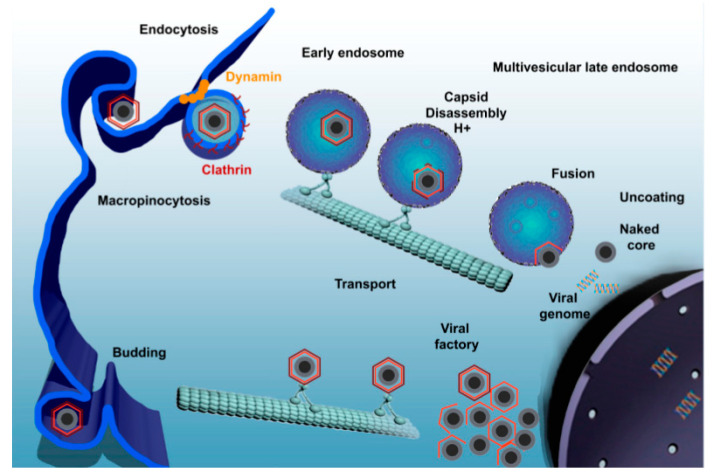
African swine fever virus (ASFV) enters the host cell through a complex process involving dynamin- and clathrin-mediated endocytosis and macropinocytosis. Newly synthesized virions are assembled in the viral factory and will exit the cell either by exocytosis budding at the plasma membrane or through the formation of apoptotic bodies [95].

Studies have shown that ASFV utilizes dynein for internalization and intracellular transport [98], entering into host cells through dynein- and clathrin-dependent endocytosis and micropinocytosis (Figure 4) [95,99]. As a microtubular motor protein, dynein is in charge of the intracellular transport linked to microtubules. In the early stage of the virus life cycle, the virus carries out intracellular transportation along microtubules. Once the virus passes through the cytoplasm, it quickly enters into the perinuclear region or nucleus and starts to replicate [100]. P54 is the main protein of the ASFV particle membrane, which can interact with the light-chain dynein of 8 kDa (DLC8) both in vitro and in cells. This interaction allows ASFV to be transported to a viral factory located in the perinuclear area at the microtubular organizing center (MTOC), which is necessary for viral protein synthesis and replication [98]. As breaking the interaction between the virus and dynein can hinder the transportation of the virus, it should be focused on as one of the mechanisms of AMPs against ASFV.

In recent years, synthetic AMPs with specific targets have been designed to bind to receptors on the surface of host cells, rendering these binding sites unavailable to viral proteins and thus impairing viral adsorption [55,101,102]. DNBLK1 is a synthesized short peptide that consists of 28 amino acid residues (Table 1) and contains DLC8 binding domain; by binding to DLC8 to prevent the interaction between ASFV protein p54 and DLC8 in vitro, it may be a useful tool to retard viral replication or spread [55]. Bruno et al. indicated that DNBLK1 can reduce the infectivity, replication, and production of ASFV, and the inhibition occurs at the early stage of the ASFV infection cycle. This provides clues for the treatment of African swine fever and other diseases caused by viruses with the same transmission mechanism as ASFV [55], and more attention should be paid to this new direction.

## 3. Conclusions

Since there is still no effective treatment for most viral infections in animal husbandry, outbreaks of viral epidemics are generally followed by the quarantine and slaughter of infected animals, resulting in great economic losses for the breeding industry and society [33,68,84,88]. In the past few decades, research on viral diseases in pig breeding has focused on the development of vaccines [5]. Vaccination can inhibit the development of the disease [40], but with the continuous variation of viruses, traditional vaccines lose their effect on mutated virus strains, and the emergence of mutant strains leads to the outbreak of viral diseases. In the context of today’s highly globalized world, viral diseases spread much faster and easier than they did centuries ago [10]. We have to face this threating challenge by utilizing more feasible options.

Therefore, in addition to the usual development of vaccines as antiviral agents, it is also of great significance to exploit new strategies to combat viruses. Currently, AMPs, due to their effective antiviral activity, are the research focus in the field of new antiviral drug development and are expected to become one of the key drivers of antiviral drug development in the future. AMPs inactivate viruses by destroying the viral envelope; binding with virus surface glycoprotein; occupying specific receptors of the host cell membrane; and inhibiting viral replication, transcription, reverse transcription, expression, and release. With the improvement of bioinformatics science, more new AMPs with antiviral activity are continuously being discovered and designed. These AMPs can resist not only porcine viruses but also Newcastle disease virus [103], duck hepatitis virus [104], bovine herpesvirus 1 [105], dengue virus [106], and other human and zoonotic viruses and thus hold great importance for research and development in theory and practice. In addition, synthetic AMPs with specific targets, such as DNBLK1 (targeting the DLC8 binding domain), have also been designed, providing a new idea and tool for the development and improvement of AMPs to resist viruses [55].

Although AMPs have great potential activity against viruses, there are still some potential problems to be solved, such as higher cost of production, shorter half-life time, and poor oral absorption of AMPs, as well as the challenge of delivery systems [107]. Some AMPs have been shown to have antiviral effects in vitro against viral diseases in animal husbandry, but their antiviral activity in vivo remains to be studied and confirmed [27,50,52]. It is known that AMPs are sensitive to trypsin and other lytic factors in vivo, especially when they are administrated orally or by blood injection. In order to improve the stability of AMPs, many studies have been carried out considering the controlled site-specific release and sustained continuous release of AMPs by nanoencapsulation [108,109] and modification of high resistance to proteolysis [110], as well including unnatural or D-amino acids substitution [111] and peptide chain cyclization [112,113]. Moreover, targeting modification is also worth considering as a powerful tool to increase killing specificity to pathogens and decrease host cell toxicity of AMPs [114,115]. In addition, many mechanisms of action between AMPs and virus molecules are still unclear and need to be further studied. However, we find it reasonable to assume that the previous fruitful findings and constructive theories from antibacterial studies with AMPs in vivo and in vitro, such as those concerning the mechanism of entry into the host cell and bactericidal details, might be shared and referenced during antiviral studies; it is confirmed from previous works that AMPs enter the blood circulation through different drug delivery routes, reach various organs, and further internalize into the cells through endocytosis and micropinocytosis [116,117,118,119]. Undoubtedly, wider and deeper new findings are highly deserving of anticipation and will attract great interest due to the unique advantages of AMPs, including their high penetration into the host cell owing to their intracellular origin, their close compatibility with the host, their hypersensitive early-warning/protection response to infection, and their low drug resistance rate owing to strong penetration and multitargeting of pathogens [30,120,121,122,123,124,125]. We strongly believe and optimistically expect that with the further elucidation of the structure, expression regulation, and mechanism of action of AMPs as a whole, the factors that limit the development of AMPs will be disclosed and overcome one by one, and more new functions of AMPs will be discovered. Eventually, AMPs will be widely utilized and commercialized in animal husbandry and even in the human health industry [16,124,126].

In summary, antimicrobial peptides, which can effectively combat viruses, are highly expected to break through their special technical bottleneck in the near future; this will enable them to support the sustainable green development of the husbandry and health industries and thus promote the general upgrading of green industry in China and the world.

## Figures and Tables

**Table 1:** Antimicrobial peptides and viruses.

Peptide	Sequence	No. of Amino Acids	Virus	Secondary Structure	Reference
**Piscidin-1**	FFHHIFRGIVHVGKTIHRLVTG	22	PRV, PEDV, PRRSV, TGEV, RV	α-Helix	[35,39,40]
**Caerin 1.1**	GLLSVLGSVAKHVLPHVVPVIAEHL	25	PRV, PEDV, PRRSV, TGEV, RV	α-Helix	[40,41]
**pBD-2**	DHYICAKKGGTCNFSPCPLFNRIEGTCYSGKAKCCIR	37	PRV, PRRSV	Combine helix and beta structure	[42]
**pBD-3**	RYYCKIRRGRCAVLGCLPKEEQIGSCSVSGRKCCRKRK	38	PRRSV	Combine helix and beta structure	[43]
**Cecropin D**	WNPFKELEKVGQRVRDAVISAGPAVATVAQATALAK	36	PRRSV	α-Helix	[27,44]
**Cecropin P1**	SWLSKTAKKLENSAKKRISEGIAIAIQGGPR	31	PRRSV	α-Helix	[45,46]
**Protegrin-1**	RGGRLCYCRRRFCVCVGR	18	PRRSV	β-Strand	[47,48]
**Protegrin-4**	RGGRLCYCRGWICFCVGR	18	PRRSV	β-Strand	[49,50]
**LL-37**	LLGDFFRKSKEKIGKEFKRIVQRIKDFLRNLVPRTES	37	PRRSV	α-Helix	[51,52]
**Epinecidin-1**	GFIFHIIKGLFHAGKMIHGLV	21	FMDV	α-Helix	[53,54]
**DNBLK1**	RRRRRRRRHPAEPGSTVTTQNTASQTMS	28	ASFV	--	[55]
